# Editorial: Emerging talents in: sleep disorders

**DOI:** 10.3389/fneur.2023.1259390

**Published:** 2023-08-07

**Authors:** Thomas Chacko Thannickal, Hiroshi Kadotani

**Affiliations:** ^1^Department of Psychiatry and Biobehavioral Sciences, David Geffen School of Medicine, University of California, Los Angeles, Los Angeles, CA, United States; ^2^Neurobiology Research, VA Greater Los Angeles Healthcare System, North Hills, CA, United States; ^3^Department of Psychiatry, Shiga University of Medical Science, Otsu, Japan

**Keywords:** sleep disorders, insomnia, anxiety disorder, sleep apnea, portable monitor, cancer and sleep

Sleep disorders are a global challenge. The causes of sleep deficiency are extremely widespread. Research shows the importance of sleep for health. The human brain is designed to function on 24-h cycles (known as circadian rhythms). These are mainly controlled by light and darkness. Good sleep is important for maintaining good health and attaining longevity. Poor quality sleep can easily compromise health and shorten one's life span. The importance of sleep for health is increasing. Sleep and circadian rhythm disorders are most common in neurodegenerative diseases.

The Research Topic “Emerging Talents in Sleep Disorders” is dedicated to highlighting students and early-career researchers working in this field and bringing exciting new ideas to it. However, many of these ideas are not communicated to a wider audience. We recognize that this is because many students and early-career researchers may lack a platform to share their ideas. This Research Topic provides them with an opportunity to succeed with their manuscripts.

Chen et al. reported the prevalence and effects of sleep-disordered breathing in middle-aged patients with sedative-free generalized anxiety disorder. Generalized anxiety disorder (GAD) and sleep-disordered breathing (SDB) share similar symptoms, such as poor sleep quality, irritability, and poor concentration during daily activities. This study investigates the proportion of undiagnosed SDB and its impact on anxiety severity and autonomic function in newly diagnosed, sedative-free GAD patients. It was found that, with an average age of 55 years and a mean BMI of 23 kg/m2, patients with GAD and matched controls had an undiagnosed SDB prevalence of approximately 50%. It was also observed that SDB correlated with worsening anxiety severity and reduced cardiac autonomic function. Importantly, age and BMI were major risk factors for predicting undiagnosed SDB.

Nakajima et al. described the transdiagnostic association between subjective insomnia and depressive symptoms in major psychiatric disorders. Comorbid depressive symptoms are associated with clinically important issues such as reduced quality of life, a poor prognosis, and increased suicide risk. This study investigated whether the association between insomnia and depressive symptoms is also found in other psychiatric disorders. In schizophrenia, a link was also found between depressive symptoms and insomnia using objective sleep assessment methods such as sleep EEG. Their results suggest that the association between subjective insomnia and depressive symptoms is a transdiagnostic feature of major psychiatric disorders.

Ding et al. examined the validation of a portable monitor compared to polysomnography for screening obstructive sleep apnea in polio survivors. Sleep-disordered breathing (SDB) is highly prevalent in polio survivors. Obstructive sleep apnea (OSA) is the most frequent type. Full polysomnography (PSG) is recommended for the diagnosis of OSA in patients with comorbidities by current practice guidelines, but it is not always accessible. The researchers investigated whether a Type 3 portable monitor (PM) or a Type 4 PM could be a viable alternative to PSG for the diagnosis of OSA in post-polio subjects. The insightful study showed that both Type 3 PM and Type 4 PM could be alternative methods to screen for OSA in polio survivors, especially for moderate to severe OSA.

Sun et al. are the authors of “Global Research on Cancer and Sleep: a bibliometric and visual analysis of the last two decades.” This systemic review analyzed the research status, hotspots, and frontiers of global research on cancer and sleep through bibliometrics and provided references and guidance for future research. Cancer and sleep disorders and the factors influencing sleep quality represent an emerging topic. This study systematically analyzed the hotspots and frontiers in the field of cancer and sleep research. The authors identified multiple hot topics for further research studies, such as intervention measures for the cancer symptom cluster, the bioavailability of exogenous melatonin, the relationship between OSA and cancer, the mechanism of tumor-induced sleep disruption, and the dose–response relationship between sleep duration and cancer risk.

Sleep problems are a growing public health concern worldwide. The National Sleep Foundation's 2023 Sleep in America survey found that almost 65% of adults who are dissatisfied with their sleep have mild or severe depressive symptoms (1). The International Classification of Sleep Disorders by the American Academy of Sleep Medicine lists 60 specific sleep-related conditions, the most common of which are insomnia, sleep apnea, restless leg syndrome, and narcolepsy (2).

The close association between altered sleep patterns and disease makes sleep a valuable diagnostic biomarker for non-invasive early diagnosis of some pathologies, as sleep disorders increase with age, and age is considered one of the major predictors of morbidity and mortality. Despite the diverse nature of sleep disorders, they have far-reaching, detrimental effects on population health, increasing both morbidity and mortality from various chronic diseases (3). The chronic effects of sleep disorders deserve more attention from clinicians, researchers, and society. In addition to a better understanding of the mechanisms of sleep, new approaches to diagnosing and treating sleep disorders are urgently needed. This editorial team thanks the reviewers for their constructive criticism.

## Author contributions

TT and HK jointly drafted and approved the final manuscript. All authors contributed to the article and approved the submitted version.

## References

[1] National Sleep Foundation. National Sleep Foundation's 2023 Sleep in America Poll. (2023). Available onine at: https://www.thensf.org/wp-content/uploads/2023/03/NSF-2023-Sleep-in-America-Poll-Report.pdf

[2] The AASM International Classification of Sleep Disorders – Third Edition. Text Revision (ICSD-3-TR). Westchester, IL: American Academy of Sleep Medicine (2023).

[3] WHO. International Classification of Diseases 11th Revision. (2022). Available online at: https://icd.who.int/en

